# Effect of diagnostic delay on survival in patients with colorectal cancer: a retrospective cohort study

**DOI:** 10.1186/s12885-016-2717-z

**Published:** 2016-08-22

**Authors:** Salvador Pita-Fernández, Luis González-Sáez, Beatriz López-Calviño, Teresa Seoane-Pillado, Elena Rodríguez-Camacho, Alejandro Pazos-Sierra, Paloma González-Santamaría, Sonia Pértega-Díaz

**Affiliations:** 1 Clinical Epidemiology and Biostatistics Research Group, Instituto de Investigación Biomédica de A Coruña (INIBIC), Complexo Hospitalario Universitario de A Coruña (CHUAC), SERGAS, Universidade da Coruña, A Coruña, Spain; 2 Surgery Department, Complexo Hospitalario Universitario de A Coruña (CHUAC), SERGAS, Universidade da Coruña, A Coruña, Spain; 3 Department of Population Screening Programs, SERGAS, Santiago de Compostela, A Coruña, Spain; 4 Department of Information and Communication Technologies, Computer Science Faculty, University of A Coruña, A Coruña, Spain; 5 Meicende Primary Care Health Centre, SERGAS, A Coruña, Spain

**Keywords:** Colorectal neoplasms, Delayed diagnosis, Survival, Mortality, Statistics, Nonparametric

## Abstract

**Background:**

Disparate and contradictory results make studies necessary to investigate in more depth the relationship between diagnostic delay and survival in colorectal cancer (CRC) patients. The aim of this study is to analyse the relationship between the interval from first symptom to diagnosis (SDI) and survival in CRC.

**Methods:**

Retrospective study of *n* = 942 CRC patients. SDI was calculated as the time from the diagnosis of cancer and the first symptoms of CRC.

Cox regression was used to estimate five-year mortality hazard ratios as a function of SDI, adjusting for age and gender. SDI was modelled according to SDI quartiles and as a continuous variable using penalized splines.

**Results:**

Median SDI was 3.4 months. SDI was not associated with stage at diagnosis (Stage I = 3.6 months, Stage II-III = 3.4, Stage IV = 3.2; *p* = 0.728). Shorter SDIs corresponded to patients with abdominal pain (2.8 months), and longer SDIs to patients with muchorrhage (5.2 months) and rectal tenesmus (4.4 months).

Adjusting for age and gender, in rectum cancers, patients within the first SDI quartile had lower survival (*p* = 0.003), while in colon cancer no significant differences were found (*p* = 0.282). These results do not change after adjusting for TNM stage.

The splines regression analysis revealed that, for rectum cancer, 5-year mortality progressively increases for SDIs lower than the median (3.7 months) and decreases as the delay increases until approximately 8 months. In colon cancer, no significant relationship was found between SDI and survival.

**Conclusions:**

Short diagnostic intervals are significantly associated with higher mortality in rectal but not in colon cancers, even though a borderline significant effect is also observed in colon cancer. Longer diagnostic intervals seemed not to be associated with poorer survival. Other factors than diagnostic delay should be taken into account to explain this “waiting-time paradox”.

## Background

Colorectal cancer (CRC) is one of the leading causes of deaths due to cancer worldwide. It is the third most common cancer in men (10.0 % of the total) and the second in women (9.2 % of the total), affecting mainly to developed regions [1]. In Europe, CRC is the most common cancer and the second most common cancer causing death, with an estimated number of 436,000 new cases and 212,000 deaths in 2008 [2].

In Spain, age-adjusted incidence rates went from 20.4 cases per 100,000 population in the period 1975–1979 to 45.9 in the period 2000–2004 [3]. Mortality registered at a much lower increase than incidence with a turning point in 1997–1998 and a subsequent decline in rates, reaching an age-adjusted mortality rate of 20.5 per 100,000 in 2003–2007 [3]. The same trend was observed in other European countries, with an increase in incidence and a decrease in mortality, probably associated with an improvement in CRC survival [4, 5].

Despite evidence clearly suggesting that CRC screening reduces CRC incidence and mortality [6], implementation of colorectal cancer screening in Spain is limited [7]. As a result, most patients with CRC are diagnosed after the onset of clinical symptoms, and only few cases are diagnosed at an asymptomatic stage as a result of screening programs.

Although symptoms vary with tumour location, typical symptoms associated with CRC include rectorrhagia, hematochezia or melena, changes in bowel habits, abdominal pain, loss of weight, iron deficiency anaemia, and intestinal obstruction [8]. Once the symptoms are presented, it is unclear the role of diagnostic and therapeutic delay in the prognosis of these patients. Intuitively, longer diagnostic delays (defined as the time between the first symptoms and the diagnosis of CRC) or therapeutic delays (defined as the time between first symptoms and initiation of treatment) might be associated with a poor prognosis. However, in regard to CRC, contradictory results have been obtained: whereas most studies have not found a significant association between delay and survival [9–14], other authors have reported, as expected, a poorer prognosis for patients with greater delays [15, 16]. Counterintuitive results have even been published, showing that patients with shorter diagnostic intervals had higher mortality rates [15, 17], leading to what is called the “waiting-time paradox”.

Two recent systematic reviews and meta-analysis have not found association between delay and CRC stage at diagnosis, nor between delay and survival [18, 19]. However, their results are not conclusive and should be interpreted taken into account the heterogeneity among the included studies, in terms of inclusion/exclusion criteria, delay definition, and the manner of measuring it. In some studies, opposite findings seem to be found in colon and rectum tumours, suggesting that future research should assess colon and rectum cancers separately.

Most of the studies have assumed a monotonic linear association between the symptom-to-diagnosis interval (SDI) and mortality [20]. To the extent that this assumption is not fulfilled, such an analysis could lead to wrong conclusions. Other authors have tested the theory of a U-shaped association between diagnostic delay and mortality in CRC patients [21–23]. Their results support the theory that longer diagnostic intervals cause higher mortality in patients with CRC. However, a higher mortality was also found in patients with very short diagnostic intervals, probably due to confounding by indication. Failure to consider this nonlinear effect may explain previous non-significant findings reported by other researchers.

In conclusion, disparate and contradictory results make studies necessary to investigate in more depth the relationship between diagnostic delay and survival in CRC. It is especially important to check the consistency of published results with that obtained from different CRC cohorts in other countries, and with different healthcare systems. Additionally, non-linear association of diagnostic delay with mortality should be investigated. The aim of this study was to determine the relationship of diagnostic delay with survival in a cohort of Spanish CRC patients.

## Methods

Observational retrospective cohort study of incident cases of CRC diagnosed at the Complexo Hospitalario Universitario A Coruña (A Coruña, northwest Spain) during 1994–2000. This is a 1382-beds public tertiary care hospital attending a population of nearly 560.000 habitants.

The study population included all incident cases with anatomopathological confirmation of CRC according to the International Classification of Diseases (ICD) 9^th^ revision (codes 153 and 154) during the study period (*N* = 1482).

This study finally included *N* = 942 patients with available data from clinical records to calculate SDI. This sample size makes it possible to detect as significant, in a Cox regression model, a relative risk of 1.3 or more associated with greater delay, assuming an exposure to this possibility of 50 % and a censored data percentage of 50 % (Security: 95 %; Statistical power: 80 %). In terms of the censoring value, we have estimated a 50 % censorship as according to published data [24] the estimated survival rate at 5 years for colorectal cancer in Spain is 61.2 %. In this situation, the sample size required to estimate a relative risk of 1.3 or more (α = 0.05, ß = 0.2) would be *N* = 912 patients.

Clinical records were reviewed retrospectively in order to collect data regarding patients’ age, gender, symptoms and signs before diagnosis, symptoms-to-diagnosis interval, location of neoplasm and TNM stage at diagnosis. Diagnosis delay or symptoms-to-diagnosis interval (SDI) was defined as the time elapsed from the date the patient perceived the first symptoms attributable to CRC until the anatomopathological confirmation of the diagnosis of cancer (date of biopsy or direct surgery). Since a patient could present more than one symptom/sign attributable to CCR before being diagnosed, the date of the earliest symptom was selected in order to calculate SDI.

Patients were followed for at least 5 years after the diagnosis, until death or censoring. Thereby, the study outcome was 5-year mortality after diagnosis. Information on death was retrieved from the Galician Mortality Registry.

### Statistical analysis

Descriptive analysis was performed for all variables studied. Continuous variables were reported using means ± standard deviations (SD) or median (interquartile range). For dichotomous/categorical variables, absolute numbers and percentages were computed.

SDI was analysed according to patients’ demographic characteristics, tumour location and stage using the Mann-Whitney and Kruskall-Wallis test. After categorizing SDI into quartiles, multivariate logistic regression analysis was performed to determine its association with the tumour stage at diagnosis, adjusting for age and gender as potential confounders.

Survival was estimated by the Kaplan-Meier method, and homogeneity of curves was assessed using the log-rank test. Influence of SDI on survival was determined in two ways. First, Kaplan-Meier survival curves were computed for each SDI interval quartile, and compared using the log-rank test. Second, SDI was treated as a continuous variable using restricted cubic splines with four knots and using the 50th percentile (3.4 months) as reference point [25]. This approach allow for a flexible association between the length of the diagnostic interval and mortality, without assuming a linear association. In both cases, the estimated 5-year hazard ratios were estimated as a function of the length of the delay interval and adjusted for age and gender, using proportional hazard Cox regression.

IBM-SPSS software, release 19 (IBM, Armonk, NY, USA) and R software v3.2.1 (The R Foundation for Statistical Computing) were used for statistical analysis. Bilateral *P*-values <0.05 were considered as statistically significant.

## Results

After inspection of clinical records, data on SDI was available in *N* = 942 (63.6 %) incident cases of colorectal cancer. Patients with missing data on diagnosis delay were similar in age (68.8 ± 11.6 vs. 68.0 ± 11.4; *P* = 0.251) and gender (58.5 % vs. 53.3 % men; *P* = 0.051) to those with accessible information.

The *N* = 942 patients with data on SDI were included in the analysis. The main characteristics of these patients were summarized in Table 1, together with the symptoms and signs evidenced before diagnosis. Mean age was 68.0 (±11.4) years, with 53.3 % being men. Most of the patients had stage II (35.0 %) or stage III (31.7 %) disease, whereas 20.1 % had metastatic stage IV colorectal cancer. Rectal bleeding was the most common symptom in tumours located in the rectum (87.0 %) and the third in colon tumours (47.1 %), behind abdominal pain (60.6 %) and changes in bowel habits (49.5 %).

**Table 1:** Features and symptoms/signs of incident cases of colorectal cancer, according with tumor’s location

	Total *N* = 942		Colon *N* = 650		Rectum *N* = 292		
	Mean ± SD	Median	Mean ± SD	Median	Mean ± SD	Median	*P*
Age, years	68.0 ± 11.4	69	68.0 ± 11.5	69	68.1 ± 11.0	68	0.922
	*N*	%					
Gender							0.236
Male	502	53.3 %	338	52.0 %	164	56.2 %	
Female	440	46.7 %	312	48.0 %	128	43.8 %	
Stage							<0.001
I	113	13.2 %	60	10.0 %	53	20.9 %	
II	300	35.0 %	226	37.5 %	74	29.1 %	
III	271	31.7 %	194	32.2 %	77	30.3 %	
IV	172	20.1 %	122	20.3 %	50	19.7 %	
Unknown	86	-	48	-	38	-	
Symptoms/signs before diagnosis^a^							
Rectal bleeding	560	59.4 %	306	47.1 %	254	87.0 %	<0.001
Change in bowel habits	489	51.9 %	321	49.5 %	168	57.5 %	0.022
Abdominal pain	454	48.2 %	393	60.6 %	61	20.9 %	<0.001
Constitutional syndrome	454	48.2 %					
Rectal tenesmus	163	17.3 %	59	9.1 %	104	35.6 %	<0.001
Anemia	126	13.4 %	107	16.5 %	19	6.5 %	<0.001
Bowel obstruction	124	13.2 %	115	17.7 %	9	3.1 %	<0.001
Abdominal mass	57	6.1 %	51	7.9 %	6	2.1 %	0.001
Mucorrhage	48	5.1 %	30	4.6 %	18	6.2 %	0.320
Rectal pain	38	4.0 %	7	1.1 %	31	10.6 %	<0.001
Bowel perforation	29	3.1 %	26	4.0 %	3	1.0 %	0.014
Intra-abdominal abscess	16	1.7 %	14	2.2 %	2	0.7 %	0.106
Rectovaginal fistula	4	0.4 %	3	0.5 %	1	0.3 %	0.999
Hematuria	3	0.3 %	3	0.5 %	0	0 %	0.556
Fecal urgency	2	0.2 %	1	0.3 %	1	0.2 %	0.525
Fecal incontinence	2	0.2 %	1	0.3 %	1	0.2 %	0.525
Anal fistula	2	0.2 %	1	0.3 %	1	0.2 %	0.525
Fecaluria	1	0.1 %	1	0.2 %	0	0 %	0.999

*SD* Standard Deviation

^a^More than one symptom or sign could be registered before diagnosis for the same patient

Median SDI was 3.4 months, significantly higher in rectum than colon tumours (3.7 vs. 3.2 months; *P* < 0.001). No significant differences were found in diagnosis delay according with gender (3.2 vs. 3.7 months; *P* = 0.051) or age (*P* = 0.100), although higher delays were found in younger patients (Table 2). Also, SDI was not found to be significantly associated with stage at diagnosis (Stage I: 3.6 months, Stage II-III: 3.4 months, Stage IV: 3.2 months; *P* = 0.728), even after adjusting for age and gender (Table 3). The same results are obtained after taking into account the diagnosis period. The symptom associated with a shorter SDI was abdominal pain (2.8 months). On the contrary, those symptoms associated with a longer delay were muchorrhage (5.2 months), rectal tenesmus (4.4 months) and rectal bleeding (4.0months) (Table 2).

**Table 2:** Symptoms-to-diagnosis interval, according to different variables

	Symptoms-to-diagnosis interval (months)		
	Mean ± SD	Median	Interquartilic range
Total	5.3 ± 6.0	3.4	1.5–6.4
Age			
< 50	6.6 ± 8.5	4.1	2.0–7.9
50–60	5.2 ± 5.1	3.4	1.9–6.5
60–70	5.4 ± 5.9	3.5	1.8–6.7
70–80	5.4 ± 6.3	3.2	1.6–6.3
> 80	4.5 ± 5.1	2.7	1.1–5.4
Gender			
Men	5.0 ± 5.8	3.2	1.4–6.3
Women	5.6 ± 6.2	3.7	1.8–6.6
Tumor location			
Colon	5.0 ± 6.0	3.2	1.3–6.2
Rectum	5.9 ± 6.0	3.7	2.2–7.3
Stage			
I	5.7 ± 6.5	3.6	2–6.2
II	5.2 ± 5.6	3.4	1.4–6.7
III	5.3 ± 6.3	3.4	1.4–6.4
IV	5.0 ± 5.5	3.2	1.8–6.1
Symptoms before diagnosis^a^			
Abdominal pain	4.6 ± 5.7	2.8	1.1–6.1
Constitutional syndrome	5.5 ± 5.8	3.5	2.2–6.3
Abdominal mass	5.4 ± 5.4	3.5	2.1–6.7
Change in bowel habits	5.6 ± 6.0	3.7	2.1–6.7
Anemia	6.0 ± 5.8	3.9	2.3–7.2
Rectal pain	6.2 ± 6.4	3.9	2.1–8.7
Rectal bleeding	6.1 ± 6.4	4.0	2.1–7.4
Rectal tenesmus	6.0 ± 6.0	4.4	2.2–7.5
Mucorrhage	8.0 ± 7.6	5.2	3.0–12.0

*SD* Standard Deviation

^a^In order to compute the symptoms-to-diagnosis interval, the earliest symptom was selected for each patient

**Table 3:** Logistic regression analysis of risk of stage III/IV at diagnosis in relation to symptom-to-diagnosis interval, adjusting for age and gender

	Total			Colon			Rectum		
	*P*	OR	95 % CI	*P*	OR	95 % CI	*P*	OR	95 % CI
Age	0.113	0.99	0.98–1.00	0.045	0.99	0.97–1.00	0.841	1.00	0.98–1.03
Gender (female)	0.412	0.89	0.68–1.70	0.253	0.83	0.60–1.14	0.833	1.06	0.63–1.77
SDI quartile	0.873			0.798			0.267		
1^st^ quartile		1			1			1	
2^nd^ quartile	0.614	1.11	0.73–1.70	0.352	1.25	0.78–2.02	0.205	0.48	0.15–1.50
3^rd^ quartile	0.839	0.96	0.62–1.46	0.523	1.17	0.72–1.90	0.068	0.35	0.11–1.08
4^rd^ quartile	0.947	1.01	0.67–1.54	0.396	1.23	0.76–1.97	0.085	0.37	0.12–1.15

*OR* Odds Ratio*, CI* Confidence Interval*, SDI* Symptoms-to-Diagnosis Interval

Overall, survival probability at 1 year was 85.9 %, at 3 years 65.1 % and at 5 years 50.5 %. No differences were found in survival between rectum and colon tumours, with a 5-year survival probability of 47.3 and 51.9 %, respectively (*P* = 0.379).

**Fig. 1:**
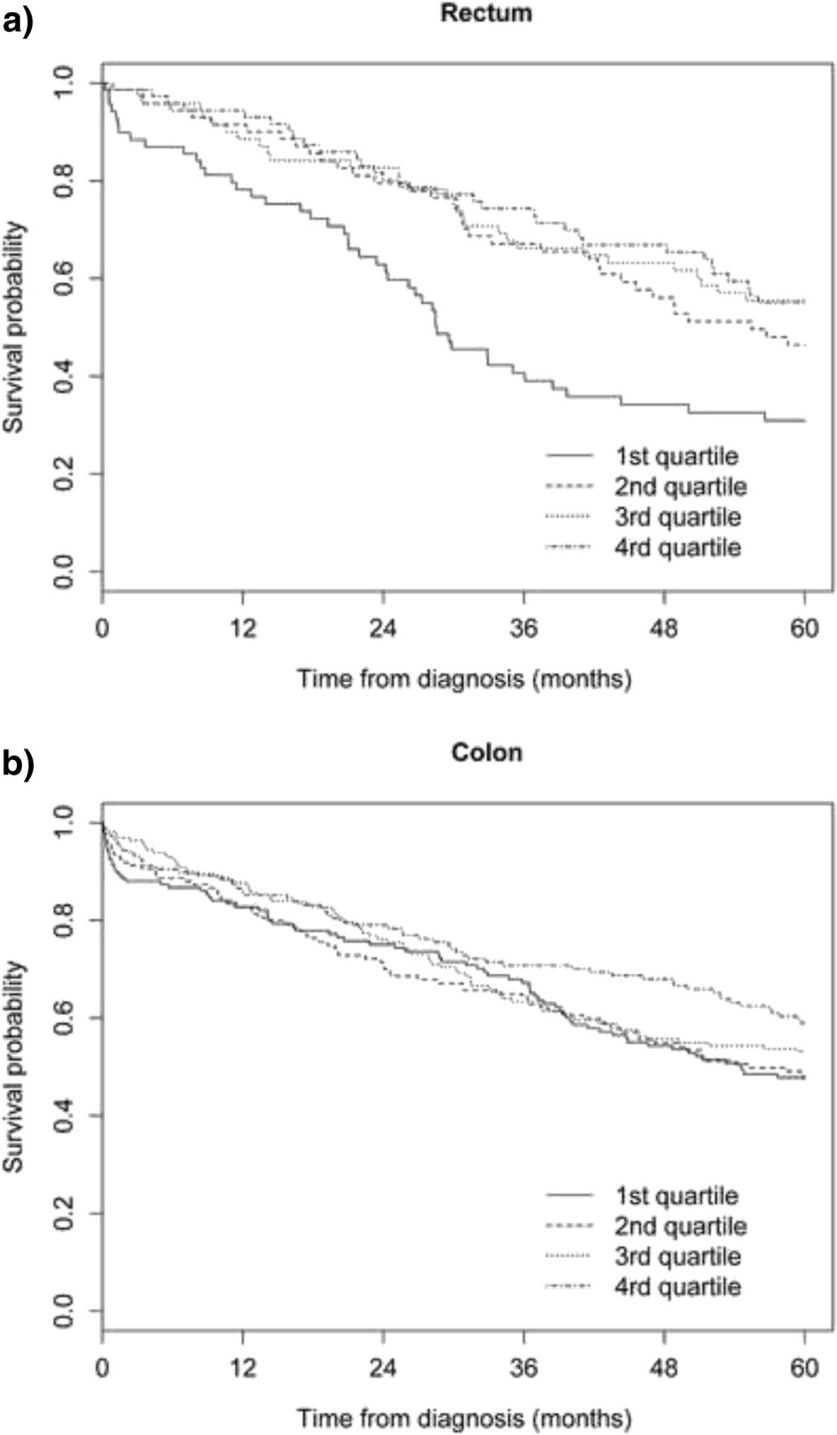
Estimated survival for each symptom-to-diagnosis interval quartile for colon and rectum incident cases

For rectal cancer patients, those with shorter delays (in the 1st quartile group of SDI) showed a significantly poorer prognosis than patients with higher delays. Therefore, survival probability at 5 years was 30.9 % for rectal cancer patients in the 1^st^ quartile group, compared with 46.5, 55.5 and 55.0 % (*P* = 0.001) for patients in the 2^nd^, 3^th^ and 4^rd^ quartile groups, respectively. On the other hand, for colon cancer patients, a progressive increase in 5-year survival was also found with SDI quartiles. Five-year survival probability increased from 47.8 % for the 1^st^ quartile group to 59.0 % in the 4^rd^ quartile group, but without reaching statistical significance (*P* = 0.163) (Fig. 1, Table 4).

**Table 4:** Estimated survival for each symptoms-to-diagnosis interval quartile for colorectal cancer incident cases

	Survival probability					
	6 months	1 year	2 years	3 years	4 years	5 years
Total						
1^st^ quartile (<1.5 months)	85.6 %	81.2 %	73.7 %	61.3 %	50.4 %	45.6 %
2^nd^ quartile (1.5–3.4 months)	90.6 %	84.1 %	71.7 %	62.9 %	52.3 %	44.2 %
3^rd^ quartile (3.4–6.4 months)	92.6 %	88.4 %	77.5 %	65.7 %	59.0 %	55.0 %
4^rd^ quartile (>6.4 months)	92.1 %	87.7 %	78.6 %	68.6 %	63.9 %	54.5 %
Log-rank = 9.263; *P* = 0.026						
Colon						
1^st^ quartile (<1.3 months)	86.7 %	82.7 %	75.1 %	67.2 %	54.3 %	47.8 %
2^nd^ quartile (1.3–3.2 months)	88.6 %	82.7 %	70.7 %	64.3 %	54.9 %	47.6 %
3^rd^ quartile (3.2–6.2 months)	92.7 %	87.7 %	76.2 %	63.4 %	55.7 %	53.1 %
4^rd^ quartile (>6.2 months)	90.5 %	85.8 %	79.0 %	70.8 %	68.0 %	59.0 %
Log-rank = 5.120; *P* =0.163						
Rectum						
1^st^ quartile (<2.2 months)	87.0 %	78.2 %	61.3 %	40.7 %	34.2 %	30.9 %
2^nd^ quartile (2.2–3.7 months)	95.9 %	91.6 %	79.5 %	67.1 %	56.1 %	46.5 %
3^rd^ quartile (3.7–7.3 months)	95.8 %	88.6 %	82.7 %	66.2 %	63.2 %	55.5 %
4^rd^ quartile (>7.3 months)	94.5 %	94.5 %	80.2 %	74.3 %	66.9 %	55.0 %
Log-rank = 16.963; *P* =0.001						

Results in the univariate analysis were confirmed after adjusting for age and gender in a multiple Cox regression model (Table 5, Model 1). Thus, a significant effect of SDI on survival was found in rectum tumours (*P* = 0.003) but not in colon tumours (*P* = 0.282). Results were similar even after adjusting for stage at diagnosis, showing that more advanced stages and lower delays are associated with a worse prognosis (Table 5, Model 2). Those results are also confirmed after taken into account the diagnosis period.

**Table 5:** Cox regression model to determine the effect of each symptom-to-diagnosis interval quartile on survival, adjusting for a) age, gender and b) age, gender and stage at diagnosis

	Total			Colon			Rectum		
	*P*	HR	95 % CI	*P*	HR	95 % CI	*P*	HR	95 % CI
a) Model 1									
Age	<0.001	1.02	1.01–1.03	<0.001	1.01	1.01–1.03	0.007	1.02	1.01–1.04
Gender (female)	0.029	0.81	0.67–0.98	0.009	0.73	0.58–0.92	0.872	1.03	0.73–1.45
SDI quartile	0.079			0.282			0.003		
1^st^ quartile		1			1			1	
2^nd^ quartile	0.979	1.00	0.75–1.34	0.798	1.04	0.76–1.43	0.022	0.59	0.38–0.93
3^rd^ quartile	0.089	0.77	0.57–1.04	0.600	0.92	0.67–1.26	0.004	0.50	0.31–0.80
4^rd^ quartile	0.104	0.78	0.59–1.05	0.115	0.76	0.54–1.07	0.001	0.46	0.29–0.74
b) Model 2									
Age	<0.001	1.02	1.01–1.03	0.001	1.02	1.01–1.03	0.003	1.03	1.01–1.05
Gender (female)	0.143	0.86	0.70–1.05	0.109	0.85	0.63–1.05	0.835	0.96	0.66–1.40
SDI quartile	0.115			0.160			0.084		
1^st^ quartile		1			1			1	
2^nd^ quartile	0.855	0.97	0.71–1.32	0.854	0.97	0.69–1.36	0.534	0.85	0.52–1.40
3^rd^ quartile	0.088	0.76	0.55–1.04	0.176	0.79	0.56–1.11	0.003	0.56	0.34–0.94
4^rd^ quartile	0.100	0.77	0.56–1.05	0.052	0.70	0.49–1.01	0.006	0.58	0.33–1.02
TNM stage at diagnosis	<0.001			<0.001			<0.001		
I		1			1			1	
II	0.002	2.07	1.29–3.33	0.083	1.72	0.93–3.18	0.003	3.23	1.51–6.92
III	<0.001	3.17	1.99–5.05	0.002	2.57	1.40–4.72	<0.001	4.94	2.38–10.24
IV	<0.001	12.40	7.56–19.81	<0.001	10.10	5.47–18.61	<0.001	19.07	9.05–40.15

*HR* Hazard Ratio*, CI* Confidence Interval*, SDI* Symptoms-to-Diagnosis Interval

**Fig. 2:**
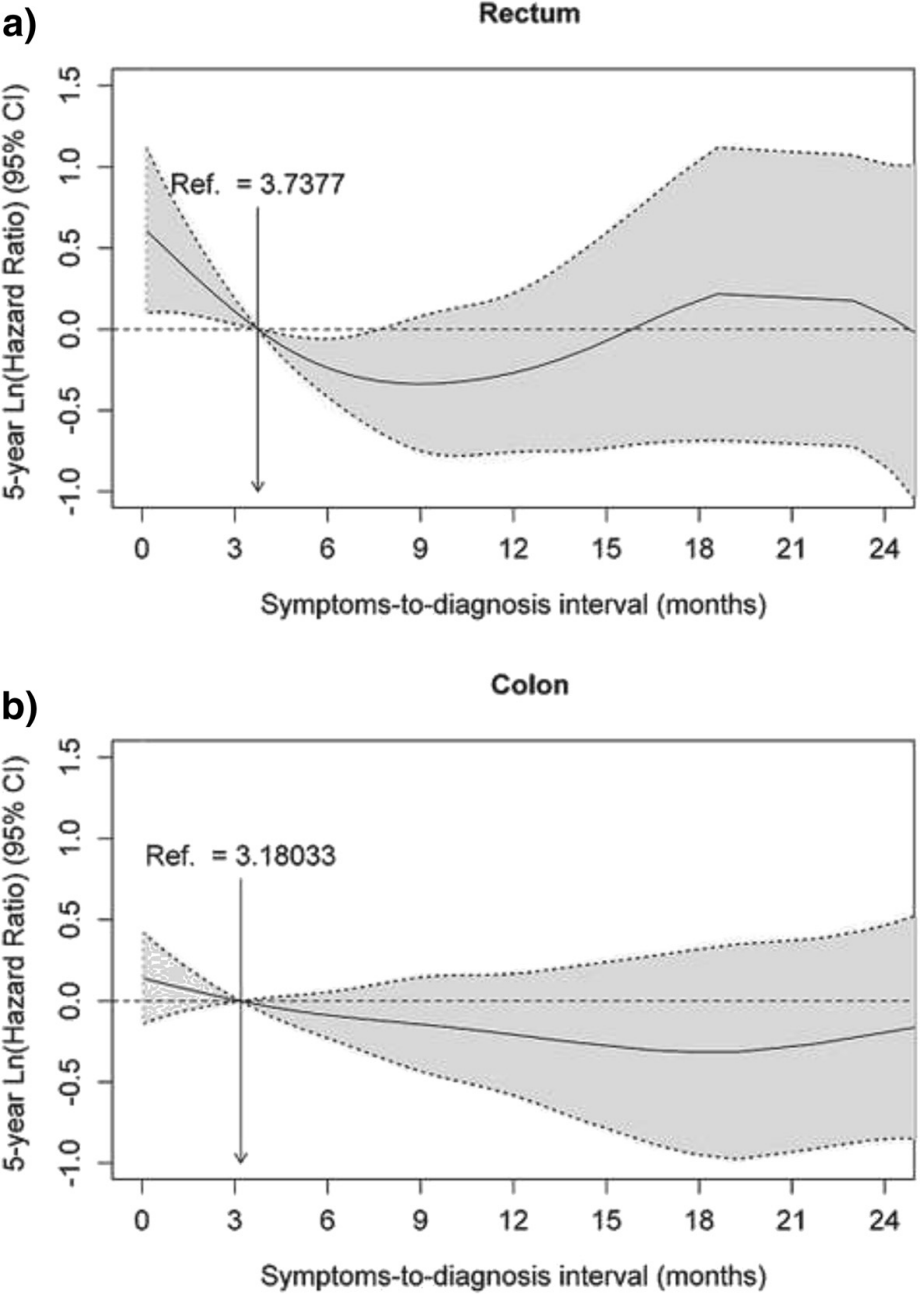
Nonparametric estimates of the dependence of overall survival on symptoms-to-diagnosis interval (restricted to the interval between 0 and 24 months) among patients with colorectal cancer (log 5-years hazard ratio, with 95 % confidence limits). Reference value = median

Furthermore, in rectal cancer patients, the cubic splines regression analysis also revealed that the 5-years adjusted risk of death decreased significantly with higher delays until approximately 7–8 months (Fig. 2a). In contrast, for colon cancer patients, no significant association was found between SDI and 5-year mortality (Fig. 2b).

## Discussion

This study examined diagnostic delay as the time from first CRC symptoms until diagnosis and assessed its association with overall mortality. A median diagnosis delay of 3.4 months was found, slightly higher in rectum than in colon tumours. Results also showed that short diagnostic intervals are significantly associated with higher mortality in rectal cancer, but not in colon cancer, even though a borderline significant effect is also observed in colon cancer. Furthermore, longer diagnostic intervals seemed not to be associated with poorer survival in colorectal cancer patients.

### Symptoms-to-diagnosis interval

Diagnostic delay in this study was significantly lower in comparison with other reported series in Spain [26, 27], being closer to the results found in a recent multicenter study made in 5 Spanish regions which reported a median delay of 4.2 months [28]. Shorter diagnosis delays were reported in studies in other countries [13, 15, 16, 22, 23]. Significantly longer SDI was found in rectal (3.7 months) than in colon (3.2 months) tumours, similarly to that reported by other authors [16, 17], although in other series a longer delay was found in colon cancers [15]. Nevertheless, comparison of delay estimates across studies is difficult because of the different definitions of delay, inclusion criteria, patients’ characteristics and disparities in models of health care delivery.

### Symptoms-to-diagnosis interval and stage at diagnosis

Results obtained also confirmed the lack of significant association between SDI and disease stage at diagnosis, which had already been reported in a recent systematic review [19]. Although most of the studies included in that review showed no significant association between delay and disease stage, a great variability was found among them, with contradictory results continuing to be published now. Therefore, while several studies find no association between diagnosis delay and stage at diagnosis [14, 16, 27], others continue to report longer delays in tumours diagnosed with earlier stages [13, 15, 17].

Although without achieving statistical signification this was also the general trend observed with our data, with median delay varying from 3.6 months for stage I to 3.2 months for stage IV tumours. However, when colon and rectal cancers were analysed separately, and after adjusting for age and gender, this trend was only observed for cancers in the rectum. The opposite was reported in the previously mentioned review [19], where a shorter delay was associated with less advanced disease in rectal cancers, while in the case of colon cancers a longer delay was associated with less advanced disease. The same finding was reported by Jullumstrø et al. [17] for colon tumours.

Therefore, although results published seems to confirm that no association exists between delay and disease stage at diagnosis, so controversy persists. Our work overcomes some of the limitations pointed out by Ramos et al. [19] for this kind of study, since it includes a large sample of incident CRC patients, using the TNM staging scheme, which is the preferred classification. However, due to the retrospective design, we could not have taken into account the potential effect or other variables such as the comorbidity or the degree of tumour differentiation, that were not available from clinical records and could act as confounders in this context.

### Symptoms-to-diagnosis interval and mortality

Similarly, results on the relationship between diagnostic delay and survival are not conclusive. Although the results of a recent meta-analysis suggest that longer delay in CRC is related to improved survival, this is by no means certain [18]. Most of the published studies include restricted samples with variable definitions of diagnosis delay. Furthermore, there were well-known prognostic factors that were not accounted for. This limitations warrant further studies to clarify the role of diagnostic delay on survival of CRC patients.

Most of the published studies do not find an association between delay and survival [13, 14, 29] or they report higher survival rates in patients with longer delays [16, 17]. Since epidemiological, etiological and genetic factors suggest that colorectal cancer is not a single entity, recent studies have analysed colon and rectal tumours separately, both describing different results. For example, Jullumstrø et al. [17] showed how increasing SDI was associated with better survival in colon cancer, but not in rectal cancer. Similar results were reported in the work by Pruitt et al. [15], where colon cancer patients with the longest diagnosis delays had higher all-cause mortality, whereas in rectal cancer patients delay was not associated with survival.

Comparison among studies is difficult, not only due to differences in their design, but also in the way data was analysed. Many studies use different cut-off points to explore the influence of SDI on survival, considering an arbitrary cut-off or the median value to classify patients with “short” or “long” delays. Other studies represent SDI as a continuous, monotonic variable in a standard Cox proportional survival analysis [30]. However, as some authors have pointed out, SDI could have different, non-linear association with mortality risk [20]. Ignoring this possibility could lead to not detecting the true nature of the relationship between SDI and survival.

One of the main strengths of this study is that it allows for a continuous, non-monotonic effect of delay in mortality risk. Following this idea, several works have reported a U-shaped association between diagnostic interval and mortality in CRC [21–23]. In a prospective, population-based study in Denmark, Tørring et al. found how the risk of dying decreased with diagnostic intervals up to 5 weeks and then increased in patients with symptoms suggestive of cancer or any other serious illness, whereas this association was reverse (although not statistically significant) in patients presenting vague symptoms [21]. Analogous results were found in a more recent study [22]. The same authors analysed three population-based CRC studies in Denmark and the UK using different methods to collect information on diagnostic delay, confirming the U-shaped association with decreasing and subsequently increasing mortality with longer diagnostic intervals [22]. More recently, the same results were replicated in another cohort of colorectal cancer, as well as in patients with lung, melanoma skin, breast and prostate tumours. For all of them, very short or long diagnostic intervals were associated with increased mortality in those patients arising with alarm symptoms suggestive of cancer. On the contrary, no statistically significant association was found between the length of the diagnostic interval and mortality in patients presenting with vague symptoms [23].

Our results confirm these findings only partially, with very short diagnostic intervals being associated with higher mortality in rectal tumours, while longer diagnostic intervals were not associated to a lower survival in rectal or colon cancers. This paradox, of shorter diagnostic intervals associated with lower survival, has been explained by the effect of unmeasured confounder factors (such as phenotype, biological virulence or tumour aggressiveness). So, rapidly growing tumours are believed to present more alarming symptoms, being associated to shorter delays and, on the other hand, to a worse survival. Other authors have argued these findings as a result of confounding by indication [21–23]. In any case, the conclusion is similar: patients with short and long SDIs are inherently different, and probably there are other factors than diagnostic delay that modify the prognosis of these patients.

On the other hand, we must keep in mind that the symptomatic period of an illness is only a little portion of the total natural history of a neoplastic disease. The asymptomatic period plays an important role, as it has been demonstrated with the efficacy of screening of CRC in asymptomatic patients to reduce mortality [6].

### Limitations

Limitations of the present study could include selection bias, information bias and residual confounding.

Perhaps the main limitation of this study is its retrospective design, which makes it vulnerable to information bias from inaccurate clinical records and missing data. We can add this to the difficult of measuring time intervals in the diagnostic pathway. Prospective studies will ideally be performed in order to measure the SDI more accurately. Furthermore, patients may remember different symptoms, that can be directly or indirectly related to the disease, and therefore differently being interpreted as first symptom. Nevertheless, in three population-based studies of incident CRC patients analogous results have been found, even when different methods of identifying the date of first presentation were used [22].

Although a large cohort of all incidents CRC cases were studied, data on SDI was unavailable in 36.4 % of those patients. There were not differences between patients with available data and patients without this information regarding age and gender. For that reason we assume that missing data are independent both of observable variables and of unobservable parameters of interest and occurred entirely at random, although the existence of some selection bias could not be totally discarded. Even though there are other time intervals that could be studied, like the symptoms-to-treatment interval (the time interval from first symptom to start of treatment), the health service interval (the time interval from the first contact with health service to diagnosis), or the treatment interval (time interval from the diagnosis to start of treatment), we focused on the SDI in order to check the consistency of our results with different meta-analysis [18, 19].

Other studies have only included patients whose diagnosis general practitioners were involved, obtaining more homogeneous groups and increasing therefore internal validity. In this study, we included all the incident cases diagnosed during the study period independently from the access to the healthcare system, increasing the external validity. Residual confounding could also be present, since there could be additional confounding factors, such as tumour aggressiveness, histological type or comorbidity which were not available in the analysis.

In the same sense, controversy exists about whether or not to adjust by tumour stage when the relationship between diagnostic delay and survival is examined. Some authors argue that stage at diagnosis is a mediator or intermediate factor between delay and survival, since it is in the pathway between both variables (longer delays cause more advanced diseases, and more advanced stages are associated with poorer survival) [21]. If it is the case, adjusting for tumour stage would introduce spurious confounding. However if we adjust for stage at diagnosis, results do not change.

## Conclusions

Despite these limitations this is a large-sample study investigating the relationship between diagnostic delay and disease stage and survival in CRC patients. After CRC symptoms, diagnosis delay seems not to affect survival of patients with tumours located in the colon. For rectum cancers survival was worse for those patients with lower SDIs. The results suggest that, contrary to what might be expected, greater diagnostic delay is not necessarily associated with a worse prognosis. However, we must not forget that long delays could be associated with other adverse events, like postoperative complications, hospital stay, quality of life or other psychosocial outcomes such as anxiety.

The different results found in the literature can probably also be explained by the methodological differences among the studies and the differences in healthcare systems. Further prospective multicenter studies, involving large cohorts of patients with all confounding factors should be designed to get more information about this issue.

## Abbreviations

CRC: Colorectal cancer
ICD: International Classification of Diseases
SD: Standard deviation
SDI: Symptom-to-diagnosis interval
